# Supplementary material for: The need for blood transfusion therapy is associated with increased mortality in children with traumatic brain injury
Source: PLoS One. 2023 Jan 6;18(1):e0279709. doi: 10.1371/journal.pone.0279709 (PMC9821419; doi:10.1371/journal.pone.0279709)
Supplement: Attachment — Submitted filename: Response to Reviewers.docx [file pone.0279709.s001.docx]

**TBI-BTT PLOS ONE Reviewer comment & responses**

We are very grateful to the Editor and Reviewers for their relevant comments that substantially improved the manuscript.

Following Reviewers’ suggestions, we have made some substantial changes in the manuscript.

All comments have also been addressed in this point-by-point response and the changes are highlighted with track changes in the manuscript.

Thank you for giving us the opportunity to revise our manuscript.

Yours sincerely,

Dr. Madhuradhar Chegondi and Dr. Balagangadhar R. Totapally, on behalf of the authors

Reviewer #1: The authors present data from the KID database evaluating the relationship between traumatic brain injury and blood product transfusion. They found that among children with severe TBI (defined by need for mechanical ventilation for >96 hours), blood transfusion was associated with higher risk of mortality.

Methods
Comment1. What is meant by “all data were weight according to HCUP recommendations before analysis to calculate national estimates.” Can the authors add a bit more robust description of HCUP and how the KID database is created? Does the KID database include non children’s hospitals? One of the variable in the logistic regression model is admission to a children’s hospital. It would help provide clarity I the description of the database were more thorough

Response: We apologize for not being clear. We have added the following paragraph in the methods section to describe the HCUP and KID database

The KID contains hospital discharge information from U.S. hospitals that participate in HCUP. Participating institutions are defined as short-term, non-federal, general, and specialty hospitals, excluding rehabilitation hospitals. The KID incorporates information on utilization, access, charges, and outcomes related to children’s medical conditions and the procedures undertaken to treat them. Fully anonymized clinical and resource use information found in a typical discharge abstract is included. Normal newborns were sampled at a rate of 10%, while complicated newborns and other pediatric discharges (age 20 years or less at the time of admission) were sampled at a rate of 80%. Sampling weight is provided for each record so that national; estimates can be calculated.

Results
Comment 2: 1. What is meant by internal injury? Does this include blunt liver and spleen injury? Any intraabdominal or intrathoracic injury? This is an odd term to describe trauma.

Response: Thank you for raising this important question.

The Clinical Classification Software Diagnoses (CCS) and Clinical Classification Software for Procedures (CCS-PCS) codes identify diagnostic and procedure groups, respectively. CCS and CCS-PCS were created by HCUP to merge multiple individual ICD-10 CM or ICD-10 PCS codes into a smaller number of clinically meaningful categories for a more uniform and standardized coding system. ((HCUP) AfHRaQHCaUP. Introduction to the HCUP KIDS’ inpatient database. www.hcup-us.ahrq.gov: 2019). We have used CCS code (234) to identify the internal injury. The CCS code 234 includes multiple trauma codes for thoracic and abdominal injuries.

We have added this paragraph in the methods section.

We modified the internal injury as “thoracic and abdominal injury” throughout the manuscript and in the tables.

Comment 3: 2. How were total charges defined?

Response: As per HCUP, total charges include total hospital charges excluding professional charges and non-covered charges.

Comment 4: 3. Table 3 – it seems like the mortality rate for children who received a blood transfusion and had sepsis was lower than the kids in the transfusion group who did not have sepsis (22.9 vs 31.8%) is this correct? Same thing with bacterial infection, mortality of 5.9% in the no infection group compared to 34.6% in the infection group.

Response: Thank you for the comment.

We rechecked our data. Yes, the mortality rate among children who received BTT and had bacterial infection and sepsis were lower compared to children who didn’t have either of them. This may be likely related to the severity of the TBI and the length of hospital stay. It appears that those who survived and stayed longer in the hospital had a higher risk of having sepsis and bacterial infections.

Comment 5: 4. Subset analysis of BTT patients – the authors report a median length of stay of 21.1 vs 3.2 days (range 2.8-3.6 days) for survivors vs those who died. I thought the inclusion criteria for the study were mechanical ventilation for a minimum of 96 hours. How can the median length of stay be less than the minimum hours of ventilation needed for study inclusion?

Response: We want to thank the reviewer for pointing out this important discrepancy in our manuscript. We completely agree with the reviewer's statement.

We have reviewed our data for the selection of cases. We have used CCS procedure code 216 for ventilation (which includes all codes for ventilation and intubation). Intubation and ventilation for operating room procedures are not included. We have updated our methods section and other relevant areas and added the description of CCS terminology. We apologize for our oversight for not updating the methods. We have verified the data for the accuracy of values presented in the manuscript. The total number of children in the KID database 2012 and 2016 with intracranial injury and ventilation (CCS code 216) are 17,199.

Comment 6: 5. The description of the model is difficult to interpret. As a clinician, what does it mean that the model correctly classified 86.4% of cases or the model explained 33% of variance? How can a clinician use this information.

Response: Thank you for the comment.

The description gives information about the robustness of the model and is useful for comparing the model with a new dataset in future research work. The 33% variance means the prediction of outcome is better by 33% compared to a flip of a coin. We are more than willing to delete the description if it is unhelpful information in a clinical manuscript.

Discussion
Comment 6: 1. I was expecting the discussion to spend more time walking the reader through the logistic regression but the discussion really feels entirely separate from the results that are presented. The manuscript is not very cohesive. Instead the discussion seems more of a series of statements about the published literature instead of a thoughtful critique of how a practicing surgeon can understand and use the data presented in the manuscript.

Response:

Thank you for the suggestion. In response to the reviewer’s suggestion, we have revised the entire discussion section. Focused on the regression and propensity score matching analyses and deleted other discussion not directly related to mortality and blood transfusion therapy.

Comment 7: 2. Why bring up trauma induce coagulopathy? It comes as a non sequitur in the discussion.

Response: We apologize for not being clear on trauma-induced coagulopathy.

Although our study objective was to evaluate the effect of PRBC in TBI, a significant number of patients in our cohort also received other blood products such as platelets, FFP, and cryoprecipitate. Which is likely explained by trauma-induced coagulopathy (TIC). By discussing the TIC, we are trying to highlight the severity of TBI in our cohort.

Per your suggestion, we deleted this paragraph in the discussion.

Comment 8: 3. Please define PRISM

Response: We expanded the PRISM acronym as follows

“Pediatric risk of mortality”.

Reviewer #2: Dear authors, congratulations on your work. I have some minor points to discuss:

Comment 1: - In the logistic regression the presence of internal injuries had a protective effect on mortality ... I found it a bit odd. Was there any interaction between variables that could explain these results? Or how do you interpret it ?

Response: Thank you for the pertinent comment.

The adjusted mortality in our model was surprisingly lower when internal injury was present. A major interaction was with APRDRG severity of illness (SOI). When we ran the model after the exclusion of APRDRG severity of illness, the adjusted odd ratio of mortality with internal injury was higher at 1.21 (95% CI: 1.1-1.3, p<0.001). The adjusted mortality with blood transfusion increased to 2.13 (95% CI: 1.9-2.4, p<0.001). However, when we tested for multicollinearity, there was no collinearity between internal injury, APRGRG SOI, and blood transfusion.

Comment 2: - In the discussion you write: "Although the overall sepsis incidence is lower in pediatric TBI [22], blood transfusion is known to be associated with an increased risk of bacterial infections and other adverse effects [23].Therefore, a higher incidence of bacterial infection and septic shock among the BTT group in our study is not surprising. "This conclusion can only be drawn in the infection/sepsis occurred after blood transfusion and based on the results presented it is not clear for the reader if this is in fact the case.

Response: Thank you so much for your comment.

Per your suggestion, given the lack of clarity we deleted this in the discussion.

Comment 3: - In the conclusion you write that "Although, our study findings favor restrictive transfusion strategy, additional studies are warranted to support restrictive transfusion practices to mitigate the adverse effects of blood transfusions in children with TBI." I agree that restrictive approach appears to be better than a liberal strategy, however, you did not provide information regarding the trigger used to transfuse the patients (perhaps because this data was not available). If the data is available it would be interesting to show how many actually were transfused only when Hb< 7g/dl and compare the mortality rate between patients with a restricted and those with a liberal policy.

Response: Thank you so much for the valuable input.

As stated by the reviewer, the KID database does not include laboratory values. Hence, we can not determine the optimal or safe transfusion threshold from our study.
