# Supplementary material for: The need for blood transfusion therapy is associated with increased mortality in children with traumatic brain injury
Source: PLoS One. 2023 Jan 6;18(1):e0279709. doi: 10.1371/journal.pone.0279709 (PMC9821419; doi:10.1371/journal.pone.0279709)
Supplement: Attachment — Submitted filename: Response to Reviewers - R2.docx [file pone.0279709.s002.docx]

**TBI-BTT PLOS ONE Reviewer comment & responses**

We are very grateful to the Editor and Reviewers for their relevant comments that substantially improved the manuscript.

Following Reviewers’ suggestions, we have made some substantial changes in the manuscript.

All comments have also been addressed in this point-by-point response and the changes are highlighted with track changes in the manuscript.

Thank you for giving us the opportunity to revise our manuscript.

Yours sincerely,

Dr. Madhuradhar Chegondi and Dr. Balagangadhar R. Totapally, on behalf of the authors

Academic Editor

Comment 1: I miss an introduction/explanation of the TBI-MV group. What are you trying to show with this? In this group, there is no differentiation between transfusion and no transfusion.

Response: We apologize for not being clear with the explanation. In the KID database GCS score was not available to classify the severity of TBI. Hence, we chose mechanical ventilation as a surrogate marker for severity, and we classified all patients with TBI who were on mechanical ventilator were considered as severe TBI. We have included this explanation in the methods section.

Comment 2: Choosing 96 hours of ventilation as a surrogate for severe TBI seems arbitrary to me. What is the ratio for this?

Response: In the last revised version we deleted 96 hours cut off and highlighted in the track changes. In the methods section we have mentioned as following

“We included patients who were discharged from hospitals with a diagnosis of TBI with ages between 1 month to less than 21 years who were mechanically ventilated.”

Comment 3: The tables or the contents of the table are only partially clear to me and need to be presented and explained more clearly.

For example, Table 1, which is supposed to represent demographics, shows a value of 36.5 (34.6- 38.5) in the transfusion group for female gender. What does this mean? I would expect an absolute number and a real value that reflects the proportion of the group. Analogously, all other dichotomous values in the tables 1-3.

Response: Thank you for the opportunity to improve the clarity of our presentation. In response to the academic editor’s comments, we have revised the tables in the new version of the manuscript. The number of patients in each group is included in the column head and values for each variable are presented as % and 95% CI. We have added the following sentence in the table titles for Tables 1-3:

“The values are presented as percentages (95% Confidence intervals)”

We chose to present the values as % for easy visualization of proportions. However, we are more than happy to change the values to absolute numbers.

Comment 4: The column "TBI-MV" creates confusion and little added value, since column 4 gives odds ratios and p-values that presumably come from comparing the "blood transfusion" and "no blood-transfusion" groups. 
Furthermore, it is not clear to which row or subgroup the p-values refer.

Response: Thank you for the suggestion. We have deleted the TBI-MV column in Tables 1-3.

Reviewer #1:

Comment 1: The manuscript is significantly improved and I commend the authors for their efforts. There are still a few issues that have not been addressed. The definition of internal injury needs to be included in the manuscript; practicing surgeons who read this paper will want to know what this term means and why it was used.

Response: Thank you so much for your feedback. In the methods section, we have included the definition of internal injury as follows:

“Thoracic and abdominal injury resulting from blunt trauma”

Comment 2: I did not see PRISM or PIN defined in the manuscript.

Response: We expanded the PRISM acronym as “Pediatric Risk of Mortality”. In response to the reviewer’s comment, we have added the following sentence in the limitations section.

“Physiologic illness severity scores such as the Pediatric Index of Mortality (PIM) or PRISM are used for quantification of physiological status using predetermined physiologic variables to facilitate accurate mortality risk estimation. These scores were not used for risk adjustment in our study.”

Comment 3: The description of the regression is improved. I still think it would be helpful for the clinical surgeon if the authors were more explicit about what these statistics mean. What does it mean that the model correctly classified 86% of cases? Is it that the model correctly predicted mortality in 86% of cases? The writing should be so clear that a surgeon with no knowledge of statistics knows exactly how these data impact clinical practice.

Response: Thank you for the comment. We have added the following sentence in the results section of the revised manuscript.

“The model has correctly predicted survival or death in 86.4% of cases.”

Reviewer #2:

Comment: The authors have answered all my queries. Thank you and I have no further comments.

Response: Thank you so much.
